# Association Between Baseline Anti-HLA (Class I and II) and Anti-MICA Antibodies and Inflammatory Cell Infiltrates in Grafted Bone After Maxillary Sinus Floor Augmentation: An Exploratory Secondary Histological Study

**DOI:** 10.3390/life16050851

**Published:** 2026-05-20

**Authors:** Sebastian Dominiak, Marzena Dominiak, Jakub Hadzik, Michał Ciszyński, Marta Kepinska, Mirosław Banasik, Aleksandra Piotrowska, Piotr Dzięgiel, Tomasz Gedrange, Alicja Baranowska, Paweł Kubasiewicz-Ross

**Affiliations:** 1 Oral Surgery Department, Wroclaw Medical University, Krakowska 26 St., 50-425 Wroclaw, Poland; sebastian.dominiak@umw.edu.pl (S.D.); marzena.dominiak@umw.edu.pl (M.D.); jakub.hadzik@umw.edu.pl (J.H.); michal.ciszynski@umw.edu.pl (M.C.); tomasz.gedrange@umw.edu.pl (T.G.); alicja.baranowska@umw.edu.pl (A.B.); 2 Department of Pharmaceutical Biochemistry, Faculty of Pharmacy, Wroclaw Medical University, Borowska 211a, 50-556 Wroclaw, Poland; marta.kepinska@umw.edu.pl; 3 Department of Nephrology, Transplantation Medicine and Internal Diseases, Institute of Internal Diseases, Wroclaw Medical University, Borowska 213, 50-556 Wroclaw, Poland; miroslaw.banasik@umw.edu.pl; 4 Department of Human Morphology and Embryology, Wroclaw Medical University, Chałubińskiego 6a St., 50-368 Wroclaw, Poland; aleksandra.piotrowska@umw.edu.pl (A.P.); piotr.dziegiel@umw.edu.pl (P.D.)

**Keywords:** maxillary sinus floor augmentation, bone regeneration, anti-HLA antibodies, anti-MICA antibodies, photobiomodulation, allograft, xenograft, histology, osteoimmunology

## Abstract

(1) Background: The role of baseline humoral immunization in bone regeneration remains unclear. This study assessed the relationship between baseline serological immunization, graft type, photobiomodulation (PBM), and histological outcomes after maxillary sinus floor augmentation. (2) Methods: This exploratory secondary analysis included 20 adults undergoing lateral maxillary sinus lifting. Patients were allocated according to graft type (allogeneic or xenogeneic) and postoperative protocol (with or without adjunctive PBM). Before surgery, serum samples were analyzed for anti-HLA class I, anti-HLA class II, and anti-MICA antibodies. After approximately 6 months, bone core biopsies were collected. Histological evaluation focused on inflammatory cell infiltrates (ICI). (3) Results: Baseline antibody positivity was detected in 35.0% of patients for anti-HLA class I, 55.0% for anti-HLA class II, and 45.0% for anti-MICA. Histological findings were generally favorable. ICI scores were low, with 65.0% of samples scoring 0 and 35.0% scoring 1. A nominal positive correlation was observed between anti-HLA class I NBG ratio and ICI; however, this finding did not remain statistically significant after correction for multiple comparisons. Exploratory PBM subgroup estimates were directionally different but were based on very small subgroups and should not be interpreted as evidence of effect modification. (4) Conclusions: The findings suggest a possible hypothesis-generating link between baseline humoral sensitization and mild local inflammatory infiltrates, which requires validation in larger, prospectively powered studies with predefined histological and immunological endpoints.

## 1. Introduction

Bone regeneration remains one of the major challenges in oral surgery and implantology. Sufficient bone volume and quality are essential for further implant treatment and may affect the long-term stability of the therapeutic outcome. Various grafting materials have been used for this purpose, including autogenous, allogeneic, and xenogeneic grafts. Allogeneic bone is particularly attractive because of its highly osteoconductive and basic osteoinductive properties, while xenografts are considered safer in terms of disease transmission and are not associated with ethical concerns [1,2,3]. In recent years, growing attention has been paid to immunological basis of bone regeneration process. Bone healing is not driven only by osteoblasts and osteoclasts. It also involves a cascade of inflammatory cells, cytokines, stromal cells, and mediators of immune system. An early and controlled inflammatory response is necessary to initiate repair, angiogenesis, and further bone remodeling. In contrast, excessive or chronic immune activation may disturb the balance between osteogenesis and resorption, resulting in lowering of the quality or quantity of bone structure [4].

In this context, antibodies directed against histocompatibility antigens may be of particular importance. The well-studied group includes anti-HLA class I and anti-HLA class II antibodies. HLA class I antigens mainly include HLA-A, HLA-B, and HLA-C molecules. Such molecules are present on most nucleated cells. HLA class II antigens mainly include HLA-DR, HLA-DQ, and HLA-DP molecules. Their expression is associated primarily with antigen-presenting cells. Hence, both of them may reflect different patterns of previous immune exposure and different mechanisms of humoral response. In solid organ transplantation, for instance, anti-HLA antibodies are a well-established risk factor for graft rejection [5,6,7]. Although an issue of anti-HLA antibodies is well-established in the literature, their significance in bone tissue healing processes remains poorly documented. Alongside anti-HLA antibodies, attention has also been drawn to anti-MICA antibodies. Their role has been studied so far, mainly in solid organ transplantation. MICA belongs to a group of stress-induced molecules. It interacts with the NKG2D receptor, which is expressed, among others, on NK cells and T lymphocytes. It is reported that anti-MICA antibodies may be associated with poorer solid organ transplantation outcomes especially when they coexist with raised anti-HLA antibodies levels [8].

In bone tissue regeneration, the significance of anti-MICA is even less understood than that of anti-HLA antibodies. To date, there are no convincing data that clearly show whether the level of immunization before bone grafting affects the quality of newly formed bone or the intensity of the inflammatory response within the graft [5,9].

Low-level laser therapy (LLLT), now often referred to as photobiomodulation (PBM), is considered a potential method of postoperative healing. At the bone tissue level, it may enhance osteogenesis and angiogenesis. Contemporary reports indicate that PBM increases the osteoblastic differentiation of multipotent cells and their further proliferation [10,11,12]. An important aspect in case of our study is immunomodulatory effect of PBM. In light of contemporary osteoimmunology, proper bone healing requires an undisturbed transition from the early pro-inflammatory phase to the reparative phase. Once disturbed, it may promote fibrosis and result in poorer quality of the grafted bone. Recent reports suggest that PBM may modulate the activity of macrophages and other immune cells. It may reduce excessive production of pro-inflammatory cytokines and promote a more pro-regenerative healing microenvironment [13,14].

The aim of the present study was to evaluate the relationship between baseline anti-HLA and anti-MICA immunization and inflammatory changes within augmented bone tissue after maxillary sinus floor augmentation, with additional assessment of the potential modifying role of photobiomodulation (PBM).

## 2. Materials and Methods

### 2.1. Study Design

**Figure 1:**
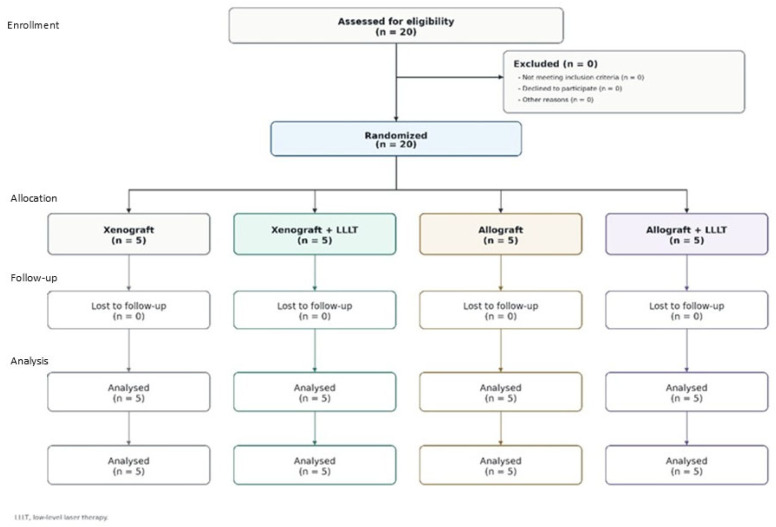
CONSORT flow diagram presenting patient enrollment, randomization and allocation to study groups, follow-up, and analysis.

The present study was designed as an exploratory secondary analysis of the same prospective randomized clinical trial and patient cohort described in our previously published report [15]. The scientific rationale and added value of the present report should be distinguished from the primary publication. The original study focused on clinical, radiological, and histological bone-regeneration outcomes after maxillary sinus augmentation with different biomaterials and adjunctive PBM. In contrast, the present secondary analysis addresses a separate immunological question: whether preoperative humoral sensitization, assessed by anti-HLA class I, anti-HLA class II, and anti-MICA antibody screening, is related to the local inflammatory component observed in bone-core biopsies after healing. Thus, the present manuscript does not aim to duplicate the primary regenerative outcome analysis, but to integrate baseline serological data with a local histological inflammatory endpoint that was not the central focus of the original report. Because this analysis was exploratory and based on the same randomized pilot cohort, its findings should be interpreted as hypothesis-generating. This prospective clinical study was conducted at the Oral Surgery Department, Wroclaw Medical University. The study included 20 adult patients requiring maxillary sinus floor augmentation prior to implant-supported rehabilitation in the posterior maxilla. The protocol was approved by the local Bioethics Committee of Wroclaw Medical University (Approval No. KB 10/2023N). All participants provided two forms of written consent: the first for lateral sinus-lift procedure and the second for participation in this study. The study was conducted in accordance with the Declaration of Helsinki, adhered to personal data protection regulations (GDPRs), and was registered in ClinicalTrials.gov under the identifier NCT07474857. The study was subsided from Wroclaw Medical University (no. SUBK.B040.23.051). The study followed the CONsolidated Standards Of Reporting Trials (CONSORT) checklist and flow diagram [16] (Figure 1).

### 2.2. Eligibility Criteria

Participants were enrolled consecutively from patients who presented for implant-supported prosthetic rehabilitation in the posterior maxilla at the MCIW Dental Clinic, the clinical facility of the Department of Oral Surgery at Wrocław Medical University, Poland, between 2023 and 2024. The study was designed as a single-center clinical investigation. All included patients required bone augmentation because of insufficient vertical bone height in the posterior maxillary region before implant placement.

A total of 20 patients indicated for maxillary sinus floor augmentation using the lateral window approach was included. Recruitment and screening for eligibility were carried out by an investigator who did not participate in the surgical procedures (A.B.).

The inclusion criteria were as follows: age of at least 18 years and the ability to provide informed consent; partial edentulism in the posterior maxilla involving one or more missing teeth; residual alveolar bone height of less than 5 mm with ridge width greater than 7 mm at the planned augmentation site; and a minimum keratinized tissue height of 2 mm in the area of interest to allow adequate primary wound closure. These eligibility criteria are consistent with contemporary recommendations and commonly used protocols for lateral window sinus floor augmentation procedures [17].

Exclusion criteria comprised poor oral hygiene (plague index > 20%), pregnancy, previous sinus surgery in the treated region, active sinus infection or large sinus cysts, as well as systemic or local contraindications to oral surgery. These included uncontrolled systemic disorders affecting bone metabolism, immunosuppression, severe cardiovascular disease, and a history of radiotherapy in the head and neck area.

### 2.3. Randomization and Allocation

As part of the original randomized clinical trial, participants had been randomly assigned to one of four parallel study groups. Randomization was performed using a simple randomization method by an independent investigator who was not involved in the surgical treatment (A.B.). Group allocation was concealed in sealed, opaque envelopes, which were opened only after patient enrollment.

Patients were allocated in a 1:1:1:1 ratio. The four groups were determined according to two variables: the type of bone grafting material and the use or absence of adjunctive PBM. Therefore, the study followed a 2 × 2 factorial design, comparing xenogeneic versus allogeneic graft material and adjunctive PBM versus no PBM.

### 2.4. Study Groups and Intervention

Allograft groups received an allogeneic cortico-cancellous bone graft in granular form with a particle size of 0.5 mm (Biobank, Lieusaint, France), while xenograft groups received a xenogeneic cancellous bone graft in granules ranging from 0.25 to 1.0 mm (Geistlich, Wolhusen, Switzerland).

The four study groups were as follows:

G1—Xenograft
G2—Xenograft with PBM
G3—Allograft
G4—Allograft with PBM

### 2.5. Preoperative Assessment

Before surgery, all patients underwent a detailed clinical examination to confirm the indication for sinus floor elevation and to exclude possible contraindications. Radiographic evaluation was then performed using cone-beam computed tomography with a Planmeca Viso^®^ G7 system (Planmeca Oy, Helsinki, Finland).

The field of view was centered on the maxilla and measured 300 mm in width and 200 mm in height. CBCT images were analyzed using Romexis software, version 6.0. The assessment included residual bone height, the condition of the maxillary sinus, Schneiderian membrane thickening, the presence of sinus polyps, cysts or septa, and possible periapical inflammatory lesions in neighboring teeth.

All radiographic measurements were performed by an investigator who was not involved in the surgical procedures (A.B.).

### 2.6. Surgical Procedure

**Figure 2:**
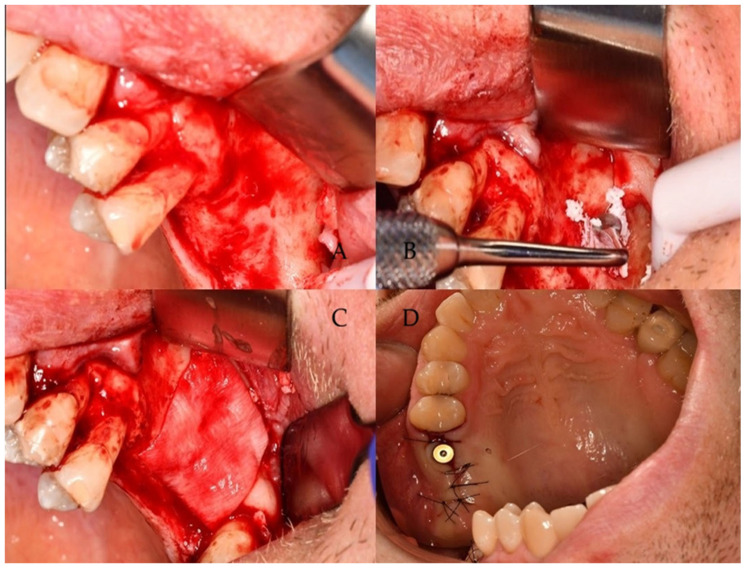
Lateral window maxillary sinus augmentation procedure. (**A**) Elevation of a full-thickness mucoperiosteal flap. (**B**) Subantral cavity filled with xenograft. (**C**) Collagen membrane placed on the lateral window. (**D**) Unsubmerged implant placed after 6 months.

All surgical procedures were carried out under local anesthesia using 4% articaine with epinephrine at a concentration of 1:200,000 (Septanest^®^ 1:200,000, Septodont, Saint-Maur-des-Fossés, France). A full-thickness mucoperiosteal flap with a distal releasing incision was raised to expose the lateral wall of the maxillary sinus. A lateral antrostomy was prepared using the DASK^®^ sinus kit (Dentium Co., Ltd., Suwon, Republic of Korea). A standardized osteotomy window was created with an 8 mm diamond drill under continuous saline irrigation and gentle pressure. The Schneiderian membrane was carefully elevated with dedicated sinus instruments to create space for the grafting material (Figure 2).

Depending on the assigned group, the sinus cavity was filled with the appropriate bone substitute. The augmented site was then covered with a resorbable collagen membrane to prevent soft tissue invasion. The amount of graft material was adjusted intraoperatively according to the size of the sinus cavity and the degree of membrane elevation; however, the exact graft volume was not recorded. Finally, the flap was repositioned and closed without tension using non-resorbable 5/0 nylon monofilament sutures.

### 2.7. Postoperative Care and Follow-Up

**Figure 3:**
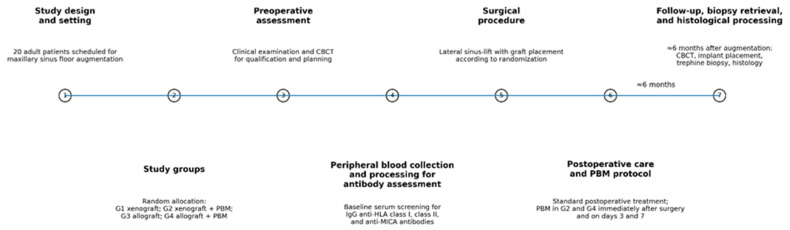
Timeline of the study protocol.

All patients received the same postoperative treatment regardless of group assignment (Figure 3). An antibiotic regimen was prescribed at a dose of 2.0 g per day, and patients were instructed to rinse with chlorhexidine mouthwash twice daily for two weeks. Sutures were removed seven days after surgery. Follow-up examinations were scheduled on postoperative days 3 and 7 to evaluate wound healing and identify any complications. Six months after surgery, CBCT imaging was performed to assess bone regeneration in the augmented area.

### 2.8. Photobiomodulation

In the groups assigned to adjunctive low-level laser therapy, photobiomodulation was performed using a diode laser (LASOTRONIX Sp. z o.o., Piaseczno, Poland) with a wavelength of 635 nm and a beam diameter of 0.5 cm^2^. The laser was applied intraorally, with the tip positioned over the osteotomy area and the surrounding soft tissues. The irradiation dose was 6 J/cm^2^, delivered continuously for one minute. The irradiation parameters were selected based on previously published photobiomodulation protocols used in sinus augmentation procedures [18]. Laser therapy was applied immediately after surgery and repeated on the third and seventh postoperative days. These parameters were selected according to standard protocols and are within the commonly reported therapeutic range for intraoral laser applications.

### 2.9. Implant Placement and Biopsy Collection

Six months after maxillary sinus floor augmentation, patients returned for implant placement. The procedure was performed under local anesthesia with 4% articaine and epinephrine 1:200,000. A full-thickness mucoperiosteal flap was raised without vertical releasing incisions. A trephine bur from the Khoury–Meisinger (Meisinger GmbH, Neuss, Germany) trephine kit was used to obtain a cylindrical bone biopsy containing both newly formed bone and residual graft particles. The biopsy sample was immediately fixed in 4% formalin and sent to the Department of Histology and Embryology at Wrocław Medical University. Bone-core biopsy retrieval at implant placement after approximately 6 months of healing is a commonly used approach in histological and histomorphometric studies evaluating sinus augmentation outcomes [19].

Implant site preparation was completed using the MIS C1 XD system (MIS Implants Technologies Ltd., Bar Lev Industrial Park, Misgav, Israel). Implant length was selected individually according to the amount of bone available after augmentation, following standard clinical guidelines. In cases with limited vertical bone height, shorter implants were preferred. When necessary, additional minor transcrestal sinus floor elevation was performed to improve primary implant stability, especially in borderline cases.

Dental implants were inserted manually using the manufacturer’s insertion instruments and a torque ratchet to ensure controlled placement. A CONNECT abutment with a healing cap was attached, and the flap was sutured around the implant to allow open healing, using non-resorbable 5/0 nylon sutures.

### 2.10. Peripheral Blood Collection and Processing for Antibody Assessment

Immediately prior to the augmentation procedure, 10 mL of peripheral venous blood was collected from the antecubital vein under aseptic conditions. Subsequently, blood was allowed to clot at room temperature for approximately 30–60 min and the sample was then centrifuged to separate serum. The serum fraction was transferred into sterile polypropylene tubes. Following this step, the material was forwarded to the Department and Clinic of Nephrology, Transplantation Medicine and Internal Diseases, Wroclaw Medical University for antibody analysis. For analytical quality, serum samples were protected from repeated freeze–thaw cycles and were stored at −80 °C. Assay setup was carried out in Luminex (Luminex Co., Madison, WI, USA) HLA antibody workflows. Baseline serum samples were screened for IgG anti-HLA class I antibodies, anti-HLA class II antibodies, and anti-MICA antibodies using a Luminex xMAP-based multiplex bead assay (DiaSorin S.p.A., Saluggia, Italy), as indicated by the pattern of variables recorded in the dataset. The test is based on color-coded microbeads coated with purified HLA and MICA antigens, followed by detection with PE-conjugated anti-human IgG. Signal acquisition is performed on a LABScan3D (Luminex Corporation, a DiaSorin Company, Austin, TX, USA) flow analyzer, and the results were interpreted using HLA Fusion software version 4.6 (One Lambda, Thermo Fisher Scientific, West Hills, CA, USA).

In the standard LABScreen workflow, 5 μL of bead mix was incubated with 20 μL of test serum for 30 min in the dark at 25 °C with gentle shaking. After washing, 100 μL of 1× PE-conjugated anti-human IgG was added and the mixture was incubated again for 30 min under the same conditions. After a further washing step, the bead suspension was read on the LABScan platform, which simultaneously identifies the bead population and measures PE fluorescence intensity corresponding to antibody binding. For result interpretation, two numerical outputs were retained from the screening assay. The first was baseline MFI, i.e., the baseline fluorescence signal derived from the antigen-coated bead after correction against the negative-control bead. The second was the NBG ratio (Normalized Background ratio), which for the LABScreen Mixed format was calculated from the sample signal corrected by the sample negative-control bead and normalized to the corresponding background-corrected negative-control serum signal.

For statistical purposes, the dataset was reduced to the maximum baseline MFI and maximum NBG ratio observed for HLA class I and HLA class II reactivity in each sample. For anti-MICA antibodies, the dataset retained the maximum NBG ratio. In addition, dichotomous variables (positive/negative) were generated from the screening readouts. According to the thresholds encoded in the study worksheet, class I reactions were considered positive at NBG ≥ 2.9, borderline at 1.7 to <2.9, and negative at <1.7; class II reactions were considered positive at NBG ≥ 3.1, borderline at 1.9 to <3.1, and negative at <1.9; the same threshold set, as applied for the class II analysis, was applied to anti-MICA. In the final analytical dataset, borderline and clearly positive reactions were collapsed into a single positive (+) category. The interpretation of MFI values and assay cut-off thresholds was performed with reference to previously published methodological recommendations for Luminex single-antigen bead assays [20].

### 2.11. Histological Processing and Evaluation

**Figure 4:**
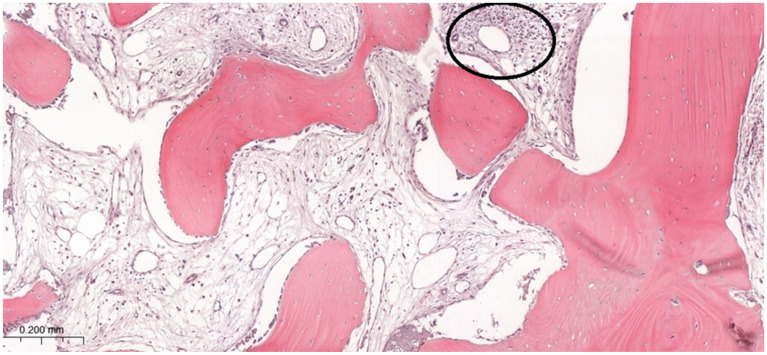
Micrograph of the grafted bone specimen. ICI indicated in the image is marked with an oval. H&E staining. Magnification ×100.

The histological slides were assessed by two independent investigators (PD and AP) who were blinded to the patients’ clinical data. The assessment was carried out using the BX-41 light microscope (Olympus, Tokyo, Japan) at 20× magnification. In case of discrepant results, the slides were reevaluated and discussed until consensus was achieved. For each slide, three representative spots (“hot-spots”) were evaluated. The biopsy material was fixed in 4% buffered formalin, decalcified in 10% EDTA, embedded in paraffin, sectioned at 4 μm, and stained with hematoxylin and eosin. Inflammatory cell infiltrates (ICIs) were identified as focal or diffuse accumulations of densely packed, basophilic cells with dark-stained nuclei located within the connective tissue, marrow spaces, or adjacent to bone trabeculae and residual graft material (Figure 4). Subsequently, the following scoring [0–3] was assigned for each specimen with the regard to number of ICI:

0 points—fewer than 1 inflammatory infiltrate per HPF (High Power Field);
1 point—fewer than 3 infiltrates per HPF;
2 points—fewer than 5 infiltrates per HPF;
3 points—fewer than 8 infiltrates per HPF.

The ICI score used in this study was a study-specific exploratory semiquantitative score and should not be regarded as a previously validated histological endpoint. However, the rationale for evaluating local inflammatory cell infiltrates in relation to baseline immunological sensitization was informed by previous studies in allogeneic intraoral bone grafting, in which systemic immune reactivity was assessed together with local histological and immunohistochemical markers of inflammation, including pro-inflammatory cytokines and T-cell markers [21]. Therefore, in the present study, the ICI score was introduced as a structured descriptive tool to characterize the presence and intensity of inflammatory cell infiltrates in the available biopsy material.

### 2.12. Statistical Analysis

Because of the small group and because a normal distribution could not be assumed, non-parametric tests were used. To assess the association between the severity of immunization and ICI outcomes, Spearman’s test was used, while the Mann–Whitney U test was applied for comparisons between two independent groups with respect to age, immunological markers, and ordinal ICI indicator. A *p*-value below 0.05 was considered statistically significant. Because several immunological markers and subgroup comparisons were evaluated, *p*-values are reported as nominal. To address multiplicity, adjusted *p*-values using the Benjamini–Hochberg procedure and Bonferroni correction were additionally calculated for the main exploratory antibody–ICI correlation analyses. Given the small sample size, these adjusted analyses were interpreted as sensitivity analyses rather than confirmatory tests.

## 3. Results

All patients completed the surgical phase of the study without complications. Adequate bone volume was achieved in every case. Consequently, all participants were qualified for implant placement and presented at the day of implantation.

### 3.1. Demographic Characteristics

The present analysis was performed on the same randomized patient cohort as that used in our previous report [15]. Overall, the study cohort included 9 men and 11 women. In the overall cohort, the mean age was 50.2 ± 10.5 years, while the median age was 49.5 years. Male participants were more frequent in G1 and G3, whereas female participants predominated in G2 and G4. The xenograft arm included 4 men and 6 women, whereas the allograft arm included 5 men and 5 women. The mean age was 44.8 ± 10.0 years in G1, 44.2 ± 7.5 years in G2, 52.4 ± 7.6 years in G3, and 59.4 ± 10.6 years in G4. Despite these numerical differences, no statistically significant differences were observed among the study groups with respect to age or sex distribution.

### 3.2. Baseline Immunological Profile

At baseline, a positive anti-HLA class I result was found in 7/20 patients (35.0%), a positive anti-HLA class II result in 11/20 patients (55.0%), and a positive anti-MICA result in 9/20 patients (45.0%). Antibody levels across the different classes did not appear to mirror one another (Table 1). This points to considerable interindividual variability in the humoral immune profile of the study population.

The immunological profile did not reach statistical significance in the main between-group comparisons (Table 2). Hovewer, several trends were visible in the raw data. Positive anti-HLA class I results were observed in 50% of the xenograft group versus 20% of the allograft group, while positive anti-HLA class II results were found in 70% and 40% of patients, respectively.

The opposite pattern was seen for anti-MICA antibodies. However, the difference was not statistically significant (*p* = 0.21 for the NBG ratio), the allograft group showed a higher mean anti-MICA NBG ratio than the xenograft group (6.38 vs. 2.28), along with a higher proportion of positive results (60% vs. 30%) (Table 3).

### 3.3. Impact of the Type of Biomaterial and PBM on the Inflammatory Process Within the Graft

Inflammatory cell infiltrates were reported in 7 out of 20 patients (35.0%). No specimens received a score of more than 1 (Table 3). No significant differences were observed between the xenograft and allograft groups for ICI (*p* = 0.68) (Table 3). Inflammatory infiltrates were more frequent in PBM groups (*p* = 0.029).

### 3.4. Influence of the Immunological Profile on the Inflammatory Process in the Graft

**Figure 5:**
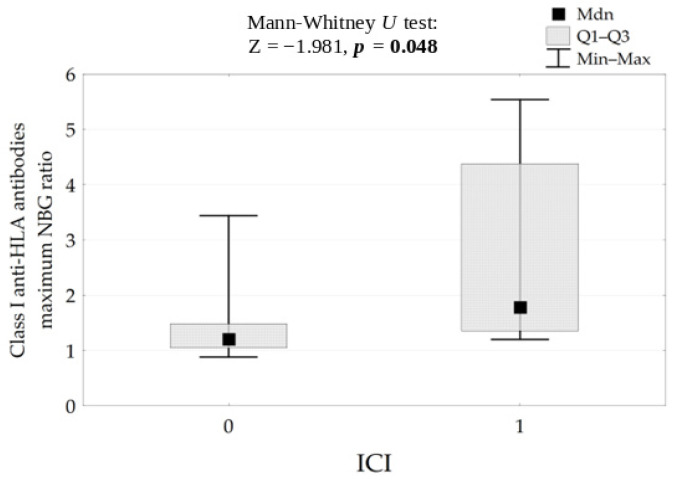
Correlation between Class I anti-HLA (NBG) antibodies level and ICI results.

A positive nominal association was observed between higher anti-HLA class I NBG ratio and higher ICI values (Spearman’s rho = 0.46; 95% CI: 0.03 to 0.75; nominal *p* = 0.048). However, after adjustment for the three exploratory antibody–ICI comparisons, this association did not remain statistically significant using either the Benjamini–Hochberg procedure or Bonferroni correction. Therefore, this finding should be interpreted as suggestive and hypothesis-generating rather than confirmatory (Figure 5).

**Figure 6:**
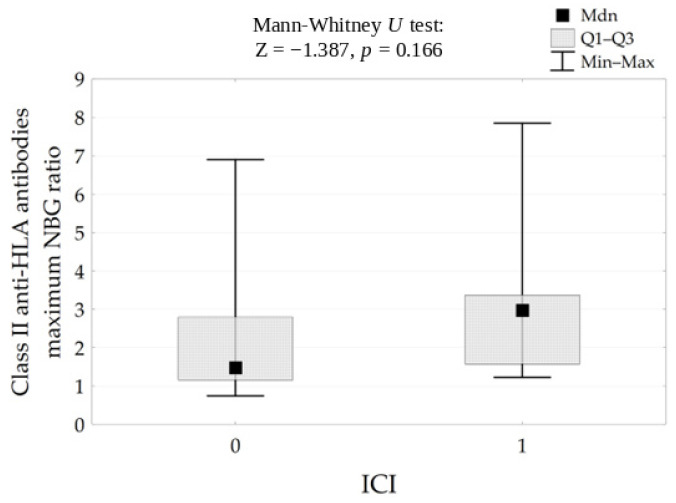
Correlation between Class II anti-HLA (NBG) antibodies level and ICI results.

For anti-HLA class II and anti-MICA antibodies, the direction of association was also positive, but the confidence intervals included zero and the results did not reach statistical significance after adjustment for multiple testing. (Figure 6 and Table 4).

**Figure 7:**
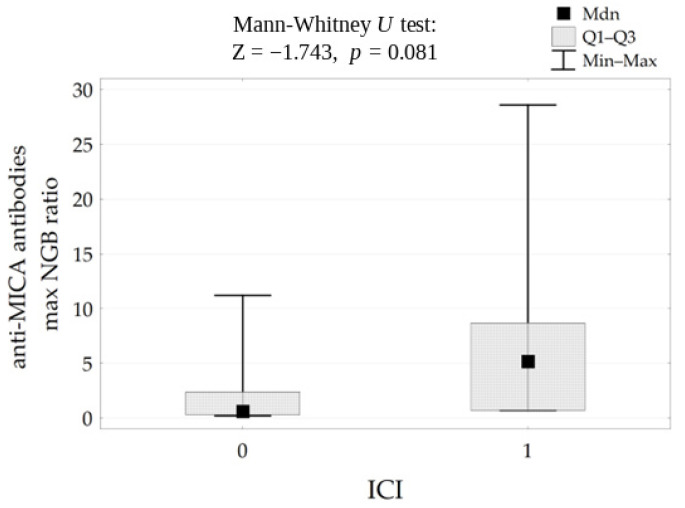
Correlation between anti-MICA (NBG) antibodies level and ICI results.

In the case of anti-MICA antibodies, the association with ICI was borderline significant (*p* = 0.081) (Figure 7).

When PBM status was considered descriptively, the correlation estimate between anti-HLA class I NBG ratio and ICI was numerically higher in the non-PBM subgroup than in the PBM subgroup. In the non-PBM subgroup, rho was 0.65 (95% CI: 0.03 to 0.91; nominal *p* = 0.044), whereas in the PBM subgroup, rho was 0.29 (95% CI: −0.42 to 0.78; nominal *p* = 0.425). Given the very small subgroup size, wide confidence intervals, and absence of formal interaction testing, these results do not provide evidence that PBM modifies the relationship between baseline immunization and ICI. They should be interpreted strictly as exploratory, hypothesis-generating observations. (Table 5).

## 4. Discussion

Although the number of patients included in this study was relatively small, it remains comparable or even larger than cohorts of similar studies involving bone biopsies following sinus augmentation. Pereira et al. reported the outcomes of 22 sinus augmentations, Pasquini et al. in randomized split-mouth clinical trial included 14 participants while Sohn et al. conducted the study on 10 participants divided into two equal subgroups [22,23,24]. It is worth noting that we found relatively high baseline immunization parameters among all participants. Having small sample size and the exploratory design in mind, it may still be cautiously assumed that the frequency of anti-HLA class I, anti-HLA class II, and anti-MICA antibodies can be relatively high in the population of people seeking regenerative procedures in the oral region.

An interpretation of significance of inflammatory cell infiltrates in regenerated bone tissue requires consideration. On one hand, a higher ICI score may reflect a more pronounced inflammatory state and may therefore be unfavorable for regeneration. On the other hand, the inflammatory phase is a physiological and necessary stage of tissue healing. In the present material, ICI values were low and limited only to scores of 0 or 1. It suggests that the link comes from small differences in graft healing, not from an obvious or excessive inflammatory response. Importantly, the type of biomaterial did not seem to play a role here.

The nominal association between anti-HLA class I reactivity and ICI should be interpreted with particular caution. Although the direction of the association was positive, this observation did not remain statistically significant after correction for multiple comparisons, and the ICI outcome had a restricted distribution limited to low-grade scores. Therefore, this result should not be interpreted as evidence of an antibody-driven inflammatory response. Rather, it represents an exploratory signal that may help formulate hypotheses for future studies. Similarly, the PBM subgroup findings should not be interpreted as demonstrating an immunomodulatory effect of PBM. The subgroup estimates were based on very small numbers, had wide confidence intervals, and were not powered to test interaction or effect modification. Therefore, these findings should be considered only as descriptive observations requiring prospective validation. These findings should be interpreted in the context of previous reports showing that the humoral response after bone grafting may be heterogeneous and may involve both anti-HLA class I and anti-HLA class II antibodies. Available studies suggest that the presence of such antibodies does not always translate into clinical failure. This remains in line with our observations. In an in vitro model, anti-HLA class I antibodies activated human microvascular endothelial cells (HMEC-1) and increased VCAM-1, ICAM-1, IL-6, CXCL8, CXCL10, and CCL5. The same study also showed increased monocyte chemotaxis and monocyte adhesion after endothelial exposure to HLA class I antibodies [25]. In kidney transplantation, clinically indicated renal biopsies from DSA (Donor Specific Antibodies)—positive patients showed 132 differentially expressed transcripts compared with DSA-negative patients, and 6 of 23 DSA-selective transcripts showed selective high expression in NK cells [26].

The literature is less consistent for anti-MICA. In heart transplantation, one study analyzed 190 pre- and post-transplant serum samples from 44 patients and examined MICA expression in 10 endomyocardial biopsies. In that cohort, anti-MICA antibodies were more frequent in severe acute rejection than in patients without rejection, with rates of 60.7% vs. 14.3% by CDC and 55.5% vs. 5.7% by Luminex [27]. Interestingly, this correlation was not found when it comes to anti-HLA antibodies. However, an opposite result was reported with regard to MICA antibodies in another heart transplant study, where MICA antibodies had no effect on acute rejection, cardiac allograft vasculopathy, or graft survival. Moreover, immunocytochemistry of cardiac biopsies from 11 patients did not demonstrate MICA expression [28].

It should be noticed that direct studies linking anti-HLA or anti-MICA antibodies with bone regeneration and bone graft histology, which was an aim of the following study, are clearly much fewer than studies in solid-organ transplantation. In the systematic review by Moraschini et al. [1], only 8 prospective human studies on the immune response to bone allografts were identified up to July 2019. In those studies, the frequency of HLA sensitization ranged from 21% to 67%, with a pooled mean of 48 ± 17.3%. It was also reported that the local and systemic consequences of this sensitization remain poorly understood [1]. In one of the few reports covering that issue, Friedlaender et al. showed that after freeze-dried bone allografts, donor-specific anti-HLA antibodies developed in 9 of 43 patients after 44 procedures. Importantly, 8 of the 9 sensitized patients who were followed radiographically had a satisfactory clinical course [29]. Later, Ward et al. reported that donor-specific HLA sensitization occurred in 57% of patients, but it had no demonstrable effect on graft incorporation or union. These studies suggest that anti-HLA antibodies may appear after bone grafting, but they do not automatically mean poor healing [7]. When it comes to reports involving oral cavity interventions, the study by de Lacerda et al. were conducted on 6 patients. All participants were treated with fresh-frozen homologous corticocancellous bone blocks. After 6 months, bone biopsies were harvested, similarly to our study, during implant placement. The authors reported 45.56 ± 15.72% vital mineralized tissue, 14.16 ± 13.39% non-vital mineralized tissue, and 40.29 ± 12.60% non-mineralized tissue. They concluded that FFHB-related HLA sensitization did not appear to affect bone incorporation, although longer follow-up is still needed [5].

The problem of local inflammatory changes in grafted area in the context of initial anti-HLA sensitization is still underinvestigated. The most evidence addressing that issue comes from broader immunologic studies of bone grafts, not from anti-HLA or anti-MICA studies alone. In a prospective randomized controlled trial, Solakoglu et al. included 60 systemically healthy participants. They used two allogeneic bone materials and one autologous control group. Bone biopsies were collected after 4 months and were analyzed for IL-1α, IL-1β, TNF-α, CD4, and CD8. The authors identified group B (*n* = 7) and group C (*n* = 5) as the immunologically more reactive subgroups. They found an association between a significant increase in IL-1α, IL-1β, and TNF-α in group C and an increase in CD4-positive cells in group B [21].

The reports that evaluate directly test effect of photobiomodulation on the immune response during regenerative bone procedures are even more limited. A recent systematic review on craniofacial bone regeneration concluded that PBMT may improve bone formation, mineralization, angiogenesis, osteoblast differentiation, and tissue remodeling. In a split-mouth clinical study of maxillary sinus augmentation with the xenograft application on 8 participants, one side was additionally treated with PBM using 660 nm wavelength. Biopsies were collected after 30 days. The PBM side showed more new bone, less soft tissue, and stronger osteocalcin staining [30]. In a rat bone-defect model study, PBM increased early expression of inflammatory and angiogenic genes at 36 h and 3 days, followed by a decrease by day 7. This suggests that PBM does not simply suppress inflammation. Instead, it appears to reshape the early inflammatory phase into a more pro-healing response [31]. Later study showed that PBMT did not increase total bone neoformation, but it did reduce polymorphonuclear cells, mononuclear cells, and osteoclast counts, while increasing the number of blood vessels throughout healing [32]. Taken together, the available original studies suggest that PBM acts more as an immunomodulator than as a dominant determinant of final bone regeneration. It may reduce excessive inflammation and improve the biologic environment for regeneration, but the number of studies directly linking PBM, immune markers, and histology of bone grafts is still small.

From a clinical perspective, the findings of the present report do not support routine preoperative anti-HLA or anti-MICA screening before sinus augmentation. However, they suggest that baseline humoral sensitization may be one of several host-related factors influencing the inflammatory microenvironment of graft healing. If confirmed in larger cohorts, antibody profiling could help identify patients who may benefit from closer postoperative monitoring or individualized regenerative protocols. Contemporary regenerative protocols increasingly combine grafting materials with biologically active adjuncts such as platelet-rich fibrin (PRF), advanced platelet-rich fibrin (A-PRF), concentrated growth factor (CGF) matrices, and photobiomodulation (PBM) to enhance angiogenesis, soft tissue healing, and modulation of the inflammatory response during bone regeneration [33,34,35].

Future studies should include larger prospectively recruited cohorts, predefined immunological and histological endpoints, and longer implant-related follow-up. Immunohistochemical characterization of inflammatory infiltrates and local cytokine profiling may help clarify whether baseline humoral sensitization affects the cellular composition and biological activity of the graft healing environment. Future studies should include larger prospectively recruited cohorts with predefined primary endpoints, including immunological, histological, and implant-related outcomes. The present findings should be interpreted as exploratory and hypothesis-generating, particularly because of the limited sample size and the semi-quantitative nature of the histological assessment. In subsequent studies, immunohistochemical or multiplex immunofluorescence characterization of inflammatory infiltrates in grafted bone specimens could be considered to better define the cellular background of the observed inflammatory response. Such analyses may include, for example, T-cell markers CD3, CD4, and CD8, B-cell marker CD20, macrophage markers CD68 and CD163, and selected NK-cell-associated markers such as CD56, CD16, or NKG2D. Assessment of these markers directly within the grafted tissue could help determine whether baseline humoral sensitization is associated not only with the intensity of inflammatory infiltrates, but also with their cellular composition and activation profile. Future research should also combine local tissue analysis with serum antibody profiling and local cytokine assessment to clarify whether anti-HLA or anti-MICA reactivity is associated with a distinct inflammatory microenvironment during graft healing. Longer implant-related follow-up, including implant survival, implant success, marginal bone stability, and peri-implant tissue health, is required to determine the clinical relevance of these findings. Finally, the potential role of photobiomodulation as a moderator of the inflammatory response should be validated in larger controlled studies stratified according to baseline immunological status.

### Limitations of the Study

The present findings should be interpreted in light of several important limitations. First, this study represents a secondary exploratory analysis of a previously published randomized pilot cohort and was not prospectively powered to assess immunological–histological associations. The total sample size was small and the original four treatment subgroups included only five patients each. Therefore, no confirmatory inference can be made from subgroup comparisons and the PBM-related findings should be interpreted only as descriptive observations rather than evidence of effect modification. Second, the ICI score was a study-specific exploratory semiquantitative scale and should not be regarded as a validated histological endpoint. In addition, all observed ICI values were restricted to scores of 0 or 1, indicating a narrow outcome distribution and limited discriminatory capacity. Third, antibody assessment was based on screening assay readouts and did not include functional antibody testing, local HLA/MICA expression analysis, cytokine profiling, or immunohistochemical characterization of the inflammatory infiltrates. Finally, the nominal association between anti-HLA class I reactivity and ICI did not remain statistically significant after correction for multiple comparisons. For these reasons, the present results should be treated strictly as preliminary, descriptive, and hypothesis-generating, requiring confirmation in larger prospectively powered studies.

## 5. Conclusions

Within the limitations of this exploratory secondary analysis, baseline anti-HLA and anti-MICA sensitization was not associated with a pronounced inflammatory response in grafted bone after maxillary sinus floor augmentation, as all inflammatory cell infiltrate scores remained low. Nevertheless, the nominal positive relationship between anti-HLA class I reactivity and low-grade inflammatory cell infiltrates suggests that humoral immune status may be a relevant host-related factor influencing the local inflammatory microenvironment during graft healing. Although this association did not remain statistically significant after correction for multiple comparisons, its consistency with the biological rationale and available immunological literature supports further investigation. The PBM subgroup findings should be interpreted cautiously, but they may indicate a potential direction for future studies evaluating photobiomodulation as a modifier of local inflammatory responses. Larger prospective studies with predefined immunological and histological inflammatory endpoints are needed to clarify the biological and clinical relevance of these observations. 

## Figures and Tables

**Table 1:** Summary of study results.

No	Class I Anti-HLA Antibodies—Max. MFI	Class I Anti-HLA Antibodies—Max. NBG	Class II Anti-HLA Antibodies—Max. MFI	Class II Anti-HLA Antibodies—Max. NBG	Anti-MICA Antibodies—Max. NBG	ICI
1	32	1.48	145	3.33	8.4	0
2	1	1.01	94	2.8	0.2	0
3	131	3.17	324	6.9	1.95	0
4	3	1.05	9	1.08	0.25	0
5	298	5.54	147	3.37	7.9	1
6	76	2.27	68	2.26	0.7	1
7	147	3.44	13	1.16	0.4	0
8	25	1.36	146	3.33	1.6	1
9	13	1.21	105	2.52	0.65	0
10	50	1.78	16	1.23	0.7	1
11	40	1.67	128	2.98	8.7	1
12	222	4.38	371	7.85	28.6	1
13	60	1.97	118	2.79	1.2	0
14	0	0.88	0	0.74	0.3	0
15	2	1.04	29	1.48	11.2	0
16	13	1.21	178	3.99	2.4	0
17	15	1.24	17	1.25	0.48	0
18	12	1.2	33	1.57	5.2	1
19	11	1.18	4	0.98	0.3	0
20	19	1.28	13	1.17	5.4	0

HLA—human leukocyte antigen; MICA—major histocompatibility complex class I chain-related antigen A; MFI—mean fluorescence intensity; NBG—normalized background ratio; ICI—Inflammatory Cell Infiltrates.

**Table 2:** Results of the statistical analysis of the baseline antibody levels.

Class of Antibody	Total *N* = 20	Xeno *N* = 10	Allo *N* = 10	*p*-Value
Class I anti-HLA antibodies—maximum baseline MFI				
*Mean* ± *SD*	58.5 ± 81.0	77.6 ± 92.8	39.4 ± 66.7	
*Mdn* [*Q*1–*Q*3]	22 [12–68]	41 [13–131]	14 [11–40]	0.23
*Min*–*Max*	0–298	1–298	0–222	
Class I anti-HLA antibodies—maximum NBG ratio				
*Mean* ± *SD*	1.92 ± 1.26	2.23 ± 1.44	1.61 ± 1.02	
*Mdn* [*Q*1–*Q*3]	1.3 [1.2–2.1]	1.6 [1.2–3.2]	1.2 [1.2–1.7]	0.23
*Min*–*Max*	0.9–5.5	1.0–5.5	0.9–4.4	
Class II anti-HLA antibodies—maximum baseline MFI				
*Mean* ± *SD*	97.9 ± 103.7	106.7 ± 94.3	89.1 ± 116.7	
*Mdn* [*Q*1–*Q*3]	81 [15–146]	100 [16–146]	31 [13–128]	0.55
*Min*–*Max*	0–371	9–324	0–371	
Class II anti-HLA antibodies—maximum NBG ratio				
*Mean* ± *SD*	2.64 ± 1.90	2.80 ± 1.71	2.48 ± 2.16	
*Mdn* [*Q*1–*Q*3]	2.4 [1.2–2.3]	2.7 [1.2–3.3]	1.5 [1.2–3.0]	0.52
*Min*–*Max*	0.7–7.9	1.1–6.9	0.7–7.9	
Class I result				
(+)	7 (35%)	5 (50%)	2 (20%)	0.35
(−)	13 (65%)	5 (50%)	8 (80%)	
Class II result				
(+)	11 (55%)	7 (70%)	4 (40%)	0.37
(−)	9 (45%)	3 (30%)	6 (60%)	

Xeno—xenograft group, Allo—allograft group, HLA—human leukocyte antigen; MFI—mean fluorescence intensity; NBG—normalized background ratio.

**Table 3:** Immunological status of the two patient groups differing in graft material and the histological analysis results in both groups.

	Total *N* = 20	Xeno *N* = 10	Allo *N* = 10	*p*-Value
anti-MICA antibodies—maximum NBG ratio				
*Mean* ± *SD*	4.33 ± 6.69	2.28 ± 3.15	6.38 ± 8.67	0.21
*Mdn* [*Q*1–*Q*3]	1.4 [0.4–6.7]	0.7 [0.4–2.0]	3.8 [0.5–8.7]	
*Min*–*Max*	0.2–28.6	0.2–8.4	0.3–28.6	
anti-MICA antibodies result				
(+)	9 (45%)	3 (30%)	6 (60%)	0.37
(−)	11 (55%)	7 (70%)	4 (40%)	
ICI (0–3)				
0	13 (65%)	6 (60%)	7 (70%)	0.68
1	7 (35%)	4 (40%)	3 (30%)	

Xeno—xenograft group; Allo—allograft group; MICA—major histocompatibility complex class I chain-related antigen A; NBG—normalized background ratio; ICI—Inflammatory Cell Infiltrates.

**Table 4:** Exploratory correlation analyses between baseline antibody reactivity and inflammatory cell infiltrates.

Immunological Marker	*n*	Spearman’s Rho	Approximate 95% CI	Nominal *p*-Value	BH-Adjusted *p*-Value	Bonferroni-Adjusted *p*-Value
Anti-HLA class I NBG ratio vs. ICI	20	0.46	0.03 to 0.75	0.048	0.122	0.144
Anti-HLA class II NBG ratio vs. ICI	20	0.33	−0.13 to 0.67	0.166	0.166	0.498
Anti-MICA NBG ratio vs. ICI	20	0.41	−0.04 to 0.72	0.081	0.122	0.243

HLA—human leukocyte antigen; MICA—major histocompatibility complex class I chain-related antigen A; NBG—normalized background ratio; ICI—inflammatory cell infiltrates; CI—confidence interval; BH—Benjamini–Hochberg procedure.

**Table 5:** Results of statistical analysis of PBM effect on ICI and anti-HLA class I association.

Subgroup	*N*	Spearman’s Rho	Approximate 95% CI	Nominal *p*-Value
Non-PBM	10	0.65	0.03 to 0.91	0.044
PBM	10	0.29	−0.42 to 0.78	0.425

PBM—photobiomodulation; CI—confidence interval.

## Data Availability

All data generated or analyzed during this study are included in this published article. No additional datasets were generated or are available beyond those presented in the manuscript.

## Abbreviations

BG	bone gain
BM	bone morphology
CBCT	cone-beam computed tomography
CCL5	C-C motif chemokine ligand 5
CONSORT	Consolidated Standards of Reporting Trials
CXCL8	C-X-C motif chemokine ligand 8
CXCL10	C-X-C motif chemokine ligand 10
DSA	donor-specific antibodies
EDTA	ethylenediaminetetraacetic acid
GDPR	General Data Protection Regulation
H&E	hematoxylin and eosin
HKT	height of keratinized tissue
HLA	human leukocyte antigen
HMEC-1	human microvascular endothelial cells
HPF	high-power field
ICAM-1	intercellular adhesion molecule 1
ICI	inflammatory cell infiltrates
IgG	immunoglobulin G
IL	interleukin
LLLT	low-level laser therapy
MFI	mean fluorescence intensity
MICA	major histocompatibility complex class I chain-related antigen A
NBG	normalized background ratio
NKG2D	natural killer group 2D
NK	natural killer
PBM	photobiomodulation
RBH	residual bone height
TB/CT	trabecular bone-to-connective tissue ratio
VCAM-1	vascular cell adhesion molecule 1
xMAP	multi-analyte profiling
